# Rapid generation of endogenously driven transcriptional reporters in cells through CRISPR/Cas9

**DOI:** 10.1038/srep09811

**Published:** 2015-04-29

**Authors:** Alejandro Rojas-Fernandez, Lina Herhaus, Thomas Macartney, Christophe Lachaud, Ronald T. Hay, Gopal P. Sapkota

**Affiliations:** 1 Centre for Gene Regulation and Expression, College of Life Sciences, University of Dundee, Dow Street, Dundee, DD1 5EH, Scotland, United Kingdom; 2 MRC Protein Phosphorylation and Ubiquitylation Unit, College of Life Sciences, University of Dundee, Dow Street, Dundee, DD1 5EH, Scotland, United Kingdom

## Abstract

CRISPR/Cas9 technologies have been employed for genome editing to achieve gene knockouts and knock-ins in somatic cells. Similarly, certain endogenous genes have been tagged with fluorescent proteins. Often, the detection of tagged proteins requires high expression and sophisticated tools such as confocal microscopy and flow cytometry. Therefore, a simple, sensitive and robust transcriptional reporter system driven by endogenous promoter for studies into transcriptional regulation is desirable. We report a CRISPR/Cas9-based methodology for rapidly integrating a *firefly* luciferase gene in somatic cells under the control of endogenous promoter, using the TGFβ-responsive gene PAI-1. Our strategy employed a polycistronic cassette containing a non-fused GFP protein to ensure the detection of transgene delivery and rapid isolation of positive clones. We demonstrate that *firefly* luciferase cDNA can be efficiently delivered downstream of the promoter of the TGFβ-responsive gene PAI-1. Using chemical and genetic regulators of TGFβ signalling, we show that it mimics the transcriptional regulation of endogenous PAI-1 expression. Our unique approach has the potential to expedite studies on transcription of any gene in the context of its native chromatin landscape in somatic cells, allowing for robust high-throughput chemical and genetic screens.

Transcriptional reporters using firefly luciferase and green fluorescent protein (GFP) have been extensively exploited to investigate the function and regulation of transcription factors. These reporters provide a robust tool for high throughput genetic and drug discovery screens^1^,^2^. Conventional reporters were constructed on a cDNA expression vector carrying either a promoter fragment or repeats of specific nucleotide sequences. In both cases, the DNA sequences are designed to recruit their cognate transcription factors followed by a minimal promoter and a gene, such as luciferase, beta lactamase or a fluorescent protein, which is used as a functional readout.

In contrast, endogenous gene promoters are positioned in a unique chromatin landscape and contain binding sites for enhancers, repressors and other cofactors, which may be located far away from the promoter^3^,^4^,^5^,^6^. As conventional transcriptional reporters contain only a fragment of the promoter region, they critically lack the chromatin context and regulatory components of the transcriptional machinery. Often they also exclude the post-transcriptional regulatory elements that exist within the non-coding regions of the mRNAs^7^. Ideally a reporter system should engage all components of an endogenous gene regulation, including the native chromatin architecture. We have achieved this by using CRISPR/Cas9 genome engineering technology^8^,^9^,^10^,^11^,^12^ to integrate a luciferase and GFP reporter cassette directly downstream of the endogenous promoter of a transforming growth factor-beta (TGFβ) responsive gene *PAI-1* (plasminogen activator inhibitor-1)^13^. TGFβ, through SMAD-transcription factors, potently induces the expression of PAI-1 transcript in many cells. Consequently, the SMAD-binding region of the PAI-1 promoter has been frequently exploited to design the conventional TGFβ-responsive luciferase reporter systems^13^. PAI-1 protein plays a critical role in the regulation of fibrinolysis by inhibiting the tissue-type and urokinase-type plasminogen activators. Polymorphisms on the PAI-1 promoter region that cause enhanced expression are associated with an increased risk of thrombosis and cancer^14^,^15^. Elevated levels of PAI-1 have also been linked to fibrotic lung disorders and development of diabetic nephropathy^16^. In contrast, decreased PAI-1 expression has been associated with lifelong bleeding disorder^17^.

The CRISPR/Cas9 genome-editing system relies on the induction of a double-strand break at the target genomic sites by the Cas9/single guide (sg) RNA nuclease complex, which recognises simple base-pair complementarity between the engineered sgRNA and its target genomic DNA sequences^8^,^9^,^10^,^11^,^12^. The CRISPR/Cas9 methodology has been used for the generation of gene knockouts and knock-ins in cells derived from a multitude of species including human, rats, mice, zebrafish, drosophila, nematodes and the parasite Plasmodium *yoelii* 18,19,20,21,22,23,24,25,26,27,28,29,30. In some cases, fluorescent proteins such as GFP have been used for the characterisation and quantification of homologous recombination events as well as for endogenous gene-tagging and determination of subcellular localisation of expressed proteins.^31^ As the detection of any fluorescent protein relies on the levels of expression, low to very low levels of fluorescent tagged proteins driven by endogenous promoters can be challenging to detect even with sophisticated tools such as confocal microscopy or flow cytometry.

## Results

**Figure 1:**
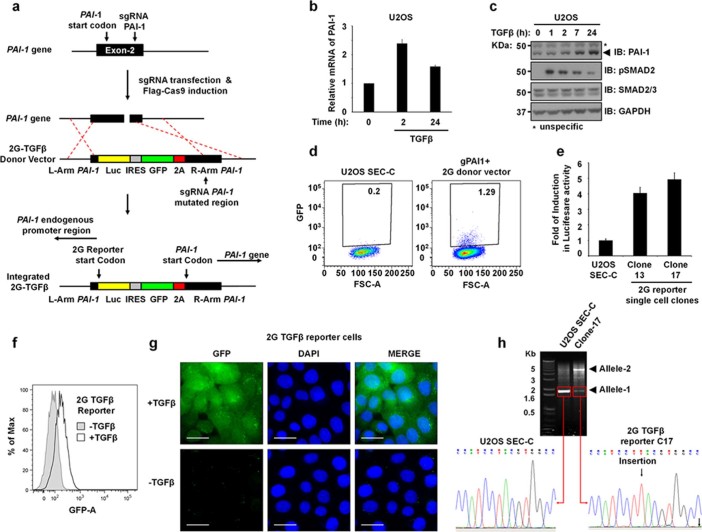
The development of an endogenously driven, second-generation luciferase and GFP reporter for the TGFβ pathway (2G TGFβ reporter). **a.** An outline of the strategy used to generate the 2G TGFβ reporter targeting the *PAI-1* gene locus. **b.** Relative expression levels of PAI-1 mRNA in WT U2OS cells stimulated with TGFβ by qRT-PCR. **c.** Western blotting of WT U2OS cells stimulated with TGFβ. **d.** U2OS(SEC-C) control and transfected (sgPAI-1 and the 2G TGFβ reporter donor vector) cells were analysed for GFP expression using FACS. **e.** Luciferase activity of GFP-expressing single cell clones was measured. **f.** Distribution of GFP fluorescence intensity in 2G TGFβ reporter cells before and after TGFβ stimulation. **g.** Wide field fluorescence microscopy imaging of GFP expression in 2G TGFβ reporter cells treated without or with TGFβ. Scale bars represent 20 μm. GFP channel indicated in green and nuclear DAPI staining in blue. **h.** EtBr-stained agarose gel showing PCR products of genomic DNA samples of U2OS(SEC-C) control and 2G TGFβ reporter (clone-17) cells. PCR products (of Allele-1) were isolated, cloned and sequenced. Clone-17 shows a frame shift insertion as indicated in chromatograms. Bar chart data are represented as mean and error bars indicate standard deviation.

In order to generate a sensitive transcriptional reporter capable of being exploited for high throughput screens, we used a firefly luciferase cassette downstream of the endogenous promoter of the PAI-1 gene. We took advantage of the non-tagged GFP downstream of luciferase to ensure fluorescence-based detection of the cassette delivery and isolation of luciferase positive clones. Using recently developed super-efficient CRISP/Cas9 system in a doxycycline-inducible osteosarcoma U2OS Stably Expressing Cas9-Cells (SEC-C),^32^ we co-transfected a vector encoding sgRNA targeting the start codon region of the PAI-1 gene (sgPAI-1) together with the TGFβ reporter donor vector containing two homologous regions (~500nts) with the PAI-1 gene, one upstream (L-Arm) and one downstream (R-Arm) of the luciferase and GFP reporter cassette, to allow for homologous recombination (Fig. 1a). To ensure the integrity of the R-Arm, we introduced silent mutations on the sgPAI-1 recognition site. The reporter cassette in the donor vector encodes the firefly luciferase enzyme cDNA, which following homologous recombination overlaps perfectly with the start codon (ATG) of the endogenous PAI-1 gene (Fig. 1a). Directly downstream of the luciferase gene, we incorporated an IRES-GFP-2a cassette. The IRES element ensures the expression of GFP separately from the luciferase protein, while the 2a self-cleaving peptide ensures the cleavage of the GFP protein from the endogenous PAI-1 further downstream, both in frame. We call this unique targeting strategy: the second-generation reporter (2G-reporter) system (Fig. 1a).

Using the U2OS parental cells, we demonstrate that the expression of both the PAI-1 transcript (Fig. 1b) and protein (Fig. 1c) are enhanced upon stimulation with TGFβ for up to 24h over basal levels. Following the transfection of U2OS (SEC-C) with sgPAI-1 and the 2G TGFβ donor vector, Cas9 expression was induced by doxycycline. After seven days, control or transfected cells were analysed by flow-cytometry (FACS) for GFP expression. Approximately 1% of the sgPAI-1 and 2G-donor transfected cell population exhibited GFP expression (Fig. 1d), indicating the integration of the 2G-reporter cassette. The efficiency of the homologous recombination is dependent on many factors, however using the U2OS SEC-C cells can yield efficiency as high as 13%, as we observed for the YFP *in situ* tagging of the Histone 3 Chaperone Chaf1a (Supplementary Fig. 4).

Single cells of the TGFβ 2G-donor GFP-expressing cells were transferred onto 96-well plates and allowed to proliferate. Of the first 33 clones tested for TGFβ responsiveness, 30 showed enhanced luciferase activity over control cells, with 28 clones showing significant induction of luciferase activity upon TGFβ treatment. Highly responsive clones were selected for further analyses (Fig. 1e). As the GFP gene is further downstream from luciferase within the 2G-donor cassette, the GFP expression in response to TGFβ was confirmed in clone-17 by measuring the percentage of maximum fluorescence in a histogram (Fig. 1f) and by fluorescence microscopy (Fig. 1g). In order to determine the genotype of the 2G-reporter clones, we amplified the genomic region of the PAI-1 gene with primers located outside of the homologous recombination arms (Supplementary Fig. 1a). All clones in this study were heterozygotes, with only one copy of the 2G-reporter cassette inserted into one of the alleles of the PAI-1 gene (Supplementary Fig. 1b). We also addressed whether the other allele was functional, by cloning the shorter amplified fragment from several clones and sequencing (Fig. 1I and Supplementary Fig. 1c). We found that the non-2G-reporter integrated alleles of PAI-1 gene were also disrupted in all TGFβ-responsive clones, including clone-17 in which there was an insertion of a single nucleotide (Fig. 1I and Supplementary Fig. 1c). Furthermore, a Southern blot analysis using a digoxigenin-labeled probe against the *firefly* Luciferase cDNA revealed a single genomic integration of the 2G donor cassette in the clone-17 (Supplementary Fig. 1E & 1F).

**Figure 2:**
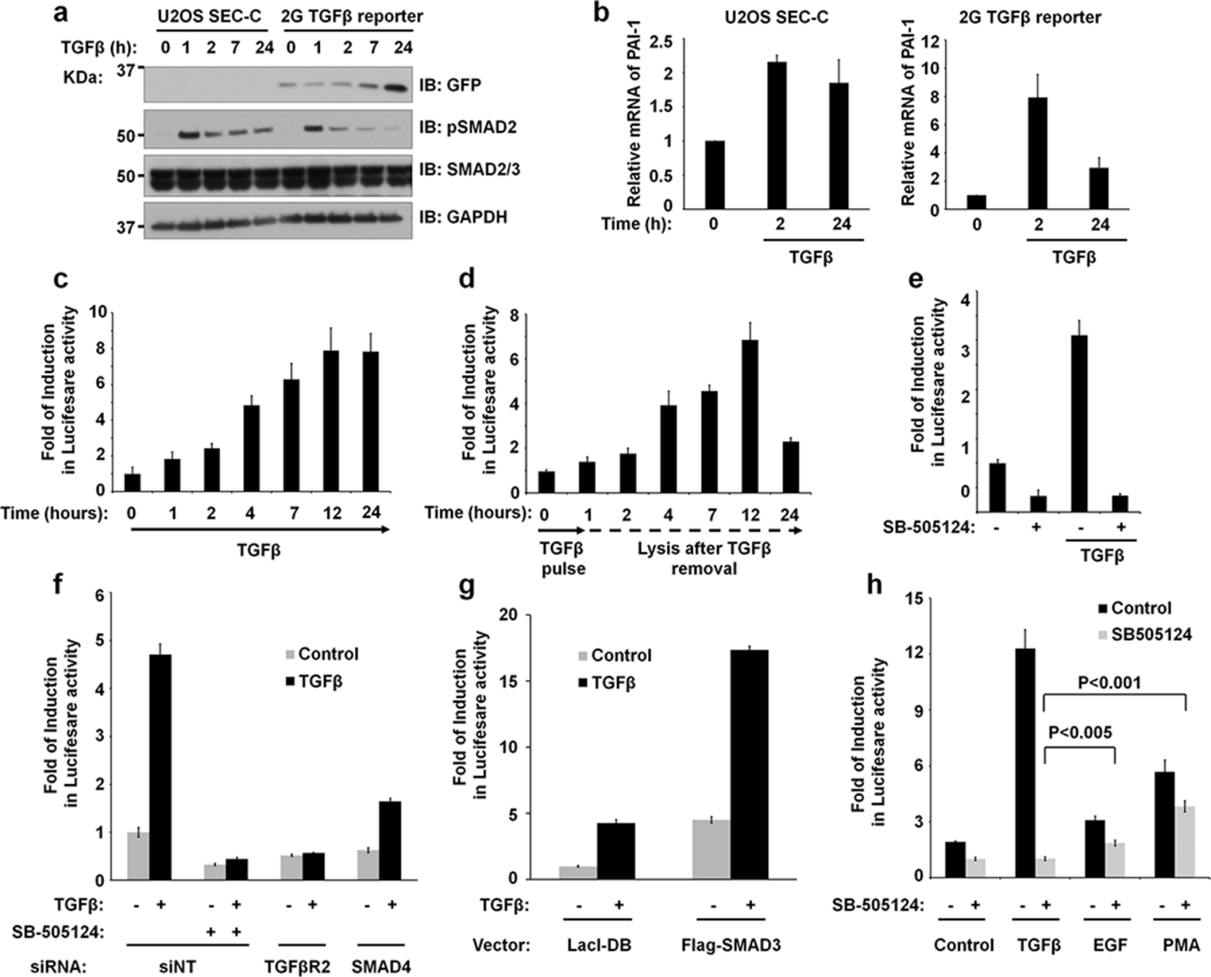
Functional characterisation of the 2G TGFβ reporter cells. **a.** U2OS(SEC-C) and 2G TGFβ reporter cells were stimulated with TGFβ for the indicated time points and lysed. The extracts were resolved and immunoblotted with the indicated antibodies. **b.** U2OS(SEC-C) and 2G TGFβ reporter cells were stimulated with TGFβ for the indicated times and the relative expression of PAI-1 mRNA analysed by qRT-PCR. **c.** 2G TGFβ reporter cells were left untreated or treated continuously with TGFβ for the indicated time points prior to lysis and luciferase activity measured. **d.** A pulse of TGFβ treatment for 1h was applied to 2G TGFβ reporter cells and upon TGFβ removal, cells were lysed at the indicated times and the luciferase activity measured. **e.** 2G TGFβ reporter cells were treated with or without TGFβ and with or without the type I TGFβ receptor inhibitor SB505124. **f.** 2G TGFβ reporter cells were transfected with siRNAs against non-targeting control (siNT), TGFβR2 or SMAD4. Cells were treated with SB505124 and/or TGFβ as indicated prior to lysis and luciferase activity measured. **g.** Luciferase activity of 2G TGFβ reporter cell extracts following transfection with vectors encoding the LacI DNA binding domain control or Flag-tagged SMAD3 (1µg per well of 12 well plates). **h.** 2G TGFβ reporter cells were stimulated with 50pM TGFβ, 50ng/ml EGF or 10 ng/mL PMA for 20h, in the presence or absence of TGFβ pathway inhibitor SB505124, prior to lysis and extracts were subjected to luciferase activity measurements. Bar chart data are represented as mean and error bars indicate standard deviation.

Next, we investigated the impact of TGFβ signalling on transcription at the PAI-1 gene locus upon integration of the 2G-reporter cassette. In U2OS(SEC-C) control cells, a time course of TGFβ induces the phosphorylation of SMAD2 (Fig. 2a). In U2OS 2G-reporter cells (clone-17), a time course of TGFβ induces similar levels of phospho-SMAD2, while GFP expression is also induced in a time-dependent manner (Fig. 2a). In these cells, as one allele of the PAI-1 gene is affected by the integration of the 2G TGFβ reporter and the insertion of a single nucleotide on other allele causes a frame-shift mutation, lower basal levels of PAI-1 mRNA are observed in comparison to U2OS(SEC-C) control cells (Supplementary Fig. 1d). Nonetheless, TGFβ induces the expression of PAI-1 transcripts in both U2OS(SEC-C) control and 2G-reporter cells (clone-17) in analogous manner over time (Fig. 2b).

A time course of sustained TGFβ activation of the 2G-reporter cells results in a time-dependent increase in luciferase activity (Fig. 2c). On the other hand, when cells exposed to a single pulse of TGFβ for 1h were lysed at various time points thereafter, the luciferase activity increased with time until it peaked at 12h and dropped significantly after 24h (Fig. 2d). Although TGFβ stimulation of the 2G-reporter cells induces robust luciferase activity and GFP fluorescence, basal detectable levels of luciferase activity and GFP expression were consistently observed under normal growth conditions. This allowed the selection of positive clones in the initial steps by cell sorting. We hypothesized that a portion of the basal levels of GFP expression and luciferase activity was the result of autocrine TGFβ signalling. Consistent with this notion, treatment of cells with SB505124, a selective inhibitor of TGFβ type I receptors, strongly inhibited the basal levels of luciferase activity (Fig. 2e). As expected, SB505124 also inhibited TGFβ-induced luciferase activity (Fig. 2e). In summary, our data demonstrates that the TGFβ-induced luciferase activity and GFP expression in 2G-reporter cells mirrors that of TGFβ-induced PAI-1 expression in other cells. Therefore, the 2G-reporter cells could be exploited as powerful tools to perform genetic and chemical screens for the identification of regulators of TGFβ signalling. As a proof-of-principal analysis, we demonstrate that siRNA*-*mediated ablation of TGFBR2 and SMAD4, which are both key transducers of TGFβ signals in cells, from 2G-reporter cells results in a decrease in both basal and TGFβ-induced luciferase activity (Fig. 2f and Supplementary Fig. 2a). On the other hand, overexpression of SMAD3, which mediates TGFβ signalling in most cells, resulted in enhanced basal and TGFβ-induced luciferase activity (Fig. 2g and Supplementary Fig. 2b).

In addition to the SMAD transcription factors downstream of TGFβ signalling, the PAI-1 promoter is reported to be regulated by several other transcription factors such as AP-1, AP-2, SP1, CTF/NF1, p53 and Elk-1^33^. The 2G TGFβ reporter cell line allows for the investigations into how PAI-1 gene expression is regulated in response to unique environmental and metabolic signals. Indeed, we demonstrate that the treatment of 2G reporter cells with either epidermal growth factor (EGF), which activates the MAPK pathway, or Phorbol 12-myristate-13-acetate (PMA), which activates AP-1 through the JNK pathway, resulted in a significant increase in luciferase activity independently of TGFβ signalling (Fig. 2h and Supplementary Fig. 2c).

## Conclusions

We describe an efficient methodology for the generation of an endogenously driven luciferase reporter at the PAI-1 gene locus in U2OS cells through the exploitation of CRISPR/Cas9 gene-editing technology. Our methodology can be exploited to rapidly integrate similar reporters on any basally or inducibly expressed genes by incorporating a fluorescent protein into a polycistronic donor cassette. Our approach using U2OS SEC-C cells allows for a highly efficient targeting of any endogenous gene^32^. In our system, the expression of Cas9 is induced directly after transfection of sgRNA by adding doxycycline. The Cas9/sgRNA-activated nuclease is further targeted to the cognate locus, thereby catalysing a DNA double strand break. In the absence of a donor vector, cells either repair this break normally or by introducing nucleotide deletions or insertions that may cause frame-shift on the gene of interest, potentially resulting in a gene knockout. In the U2OS SEC-C cells, even if the gene is repaired properly after the first break, as long as the Cas9/sgRNA are still active and present, they will continually induce breaks at the locus. Thus, we introduced a negative selection for proper repair. By adding a donor vector, we induced a homologous recombination to integrate the polycistronic cassette at the correct locus.

In exploiting our approach further, we have identified key factors that play important roles in determining the efficiency of homologous recombination. Firstly, the nature of the gene is a major factor - we observed that when we rescue the expression of an essential gene targeted by CRISPR/Cas9 by co-transfecting a donor cassette, the efficiency increases. An example is shown in the Supplementary Fig. 4, where we endogenously tagged the essential Histone H3 chaperone Chaf1a with a yellow fluorescent protein (YFP). The efficiency of YFP positive cells was over 13% compared to 1% for the PAI-1 locus. 100% of the YFP-positive cells showed at least one Chaf1a allele tagged with YFP. Furthermore, ~1% YFP-positive cells exclusively expressed the Chaf1a-YFP fusion protein indicating the presence of homozygous integration, where both alleles have been tagged. This data confirms that U2OS SEC-C cell line is a good system for using CRISPR/Cas9 technology not only for gene knockouts but also for knock-ins. Secondly, as has been previously reported, the homogeneity of the Cas9 nuclease expression also plays an important role in the efficiency of cleavage and hence that of homologous recombination^32^ in U2OS SEC-C cells, in which Cas9 expression is isogenically expressed by doxycycline. Thirdly, the properties of sgRNA also play an important role in determining the efficiency of recombination. We observed that different sets of gRNAs targeting the proximal regions of a gene transfected under identical circumstances resulted in different efficiencies of homologous recombination (data not shown).

The expression of GFP in our system allowed us to select positive clones through flow cytometry, allowing us to enrich for target cells even though the efficiency of the integration was low. It is conceivable that the insertion of an IRES results in a moderate alteration in expression of the target gene. Therefore, when a 2G reporter is used, we suggest choosing a gene that is unlikely to affect cellular physiology when its expression is moderately altered. In the case of PAI-1 gene, the protein product is secreted to the extracellular matrix, where it plays a key role in fibrinolysis but does not impact the physiology of the cells producing it. In our experiments with the polycystronic cassette, ~85% of the single isolated GFP positive clones also displayed significantly elevated luciferase activity upon TGFβ treatment, suggesting efficient integration of the cassette at the target locus. Considering that in the absence of GFP co-expression the generation of a luciferase reporter would be very laborious, we consider our system efficient and rapid. In mammalian cell lines, targeting of a second-generation reporter should take less than 3 weeks (Supplementary Fig. 3). In summary, our methodology can be exploited in gene editing strategies in cells by replacing the endogenous genes at the start codon with luciferase and fluorescent proteins, which allows for rapid selection of positive clones through FACS sorting. Remarkably the luciferase-based reporters do not require sophisticated instruments for detection and provide a sensitive and quantitative detection of transcription for high-throughput as well as small-scale experiments. Using our system, we were able to verify the relative TGFβ-dependent and independent expression of PAI-1 in cells.

## Methods

### Construction of a second generation Luciferase and GFP reporter for genome editing at the PAI-1 locus with CRISPR/Cas9

Analysis of the N-terminal coding region of PAI-1/SERPINE1 (ensembl ENSG00000106366) revealed a number of potential Cas9 guide sites in exon 2, which were identified as demonstrating high target affinity, high efficiency and a low off-target scores using the E-CRISPR software (http://www.e-crisp.org/E-CRISP/reannotate_crispr.html). The optimal scoring guide target site GCACCATCCCCCATCCTACG[TGG] (corresponding to nucleotides 72-94 of PAI-1 NM_000602.4, PAM site in brackets) was chosen to perform the CRISPR gene-editing using wild-type Cas9. This sequence was cloned into pESgRNA via site-directed mutagenesis using the QuickChange method (Stratagene) in conjunction with KOD Hot Start DNA polymerase (Novagen) and sequence verified (pESgPAI-1 vector)^32^.

An N-terminal donor was designed around the point of Cas9 cleavage site to replace the ATG start codon of PAI-1 with a polycistronic insert consisting of a Luciferase-hPEST-IRES2-GFP-2A cassette surrounded by 500 bp flanking homology arms to the locus. The insert was designed to remain in-frame with PAI-1 and the use of IRES and 2A elements would ensure the expression of both the luciferase and GFP reporters as well as the native PAI-1 gene as separate proteins. Additional silent mutations were engineered to the guide region to ensure the donor was not open to attack by the Cas9 nuclease. This second generation TGFβ reporter donor (2G TGFβ reporter) was synthesized by GeneArt (Life Technologies) (Supplementary Figure 2). All the reagents and cell lines used in this study are available to request from the MRC-PPU Reagents Page (https://mrcppureagents.dundee.ac.uk).

### Transfection of guide RNA and donor vector into U2OS SEC-C cells

A U2OS Flp-In T-REx cell line has been stably transfected with a pcDNA5 FRT-TO-Cas9-Flag vector and further selected for an isogenic population under hygromycin selection. The U2OS Stably Expressing Cas9 Cell line (SEC-C) has resulted in highly efficient Cas9-induced double strand breaks and the generation of knockout cell lines^32^. We exploited the U2OS SEC-C for the generation of the 2G TGFβ reporter. 1x10^5^ U2OS SEC-C Cas9-Flag cells were co-transfected with 3 µg of pESgPAI-1 and 3µg L-ARM (Luciferase-hPEST-IRES2-GFP-2A) R-ARM donor vectors using Lipofectamine 2000, according to the manufacturer's instructions. Next, cells were incubated for 12 h in Dulbecco’s modified Eagle’s medium (DMEM) supplemented with 10% fetal bovine serum (FBS), 2 mM L-glutamine, 100 µg/ml transfection compatible antibiotic NormocinTM (Invivogene). The medium was then replaced by fresh medium supplemented with 2 µg/ml doxycycline to induce Cas9 expression and cells were incubated for an additional 24 h. To increase the efficiency of homologous recombination, a repeat transfection on the same cells following identical procedure was carried out and cells were maintained in medium supplemented with 2 µg/ml doxycycline for 7 days, with fresh medium replaced every two days.

### Flow cytometry and cell sorting

Cells were analysed for GFP fluorescence on an LSR Fortessa or FACS Canto flow cytometer (Becton Dickinson) and data analysed using FlowJo software (Tree Star Inc). Single cells were identified on the basis of FSC-A, FSC-W and SSC-A and GFP fluorescence measured with 488 nm excitation and emission detected at 530 ± 30 nm. Cell sorting was performed on an Influx cell sorter (Becton Dickinson) with FACS Sortware, using the same cell identification procedure as described above. Single GFP expressing cells were sorted onto individual wells of a 96 well plate containing DMEM supplemented with 20% fetal bovine serum (FBS), 2 mM L-glutamine, 100 units/ml penicillin and 100 μg/ml streptomycin and 100 µg/ml transfection compatible antibiotic NormocinTM (Invivogene). Single cell clones were allowed to proliferate and analysed for luciferase activity and GFP fluorescence as described below.

### Cell culture, stimulation and lysis

U2OS cells were cultured in DMEM supplemented with 10% FBS, 2 mM L-glutamine, 100 units/ml penicillin and 100 μg/ml streptomycin. Cells were maintained at 37°C in a humidified atmosphere with 5% CO_2_. Human recombinant TGFβ1 (R&D Systems) was diluted in a solution containing 4 mM HCl and 0.1% BSA. Cells were serum starved for 16 h at 37°C prior to treatment with TGFβ1 (50 pM) for the indicated times prior to lysis. Cell lysis for immunoblotting applications was performed by washing cells once in 1 x PBS and scrapping on ice in lysis buffer (50 mM Tris/HCl pH 7.5, 0.27 M sucrose, 150 mM NaCl, 1 mM EGTA, 1 mM EDTA, 1 mM sodium orthovanadate, 1 mM sodium β-glycerophosphate, 50 mM sodium fluoride, 5 mM sodium pyrophosphate, 1% Triton X-100, 0.5% Nonidet P-40) supplemented with complete protease inhibitors (1 tablet per 25 ml) and 0.1% β-mercaptoethanol. Extracts were centrifuged for 10 min at 4°C, 14000 rpm and processed immediately or snap frozen in liquid nitrogen and stored at -80°C. The protein concentration was determined using the Bradford method^34^. siRNA SMARTpool On-Target plus mix (Dharmacon) of 4 siRNA oligos were used for knockdown experiments: non-targeting pool siNT (D-001810-10), TGFBR2 (LU-003930), SMAD2 (LU-003561) and SMAD4 (LU-003902). siRNA transfections were performed using Lipofectamine RNAiMax according to the manufacturer's instructions. For mRNA isolation, cells were processed using an RNA extraction kit according to the manufacturer’s instructions (Qiagen RNeasy kit).

### Luciferase assay

For luciferase assays, cells were lysed, after washing twice with 1xPBS, by adding passive lysis buffer (Promega) directly onto the plates following the manufacturer’s protocol. Extracts were collected and centrifuged at 14000 rpm for 5 min. 50 µl of cleared extract was transferred into each well of a 96 well plate (#655083, Greiner Bio-one) containing 50 µl of 2 x Luciferase Buffer (50 mM Tris/phosphate (pH 7.8), 16 mM MgCl2, 2 mM DTT (dithiothreitol), 30% (v/v) glycerol, 1 mM ATP, 1% BSA, 0.25 mM luciferin and 8 μM sodium pyrophosphate) and the data was obtained using an Envision 2104 plate reader (PerkinElmer). Protein concentration, determined by BCA Protein Assay Reagent (Pierce), was used for data normalisation.

### Microscopy

Cells were plated on 12 mm coverslips. After TGFβ treatment, cells were fixed for 10 min in 3.7% formaldehyde at room temperature for 20 min and permeabilised with 0.2% Triton X-100 for 5 min. Next, cells were washed 3 times with PBS and incubated for 5 min with 0.1 µg/ml DAPI. Finally, the coverslips were washed 3 times with PBS, twice with water and once with ethanol, before mounting them onto microscope slides with Vectashield (Vector H-1000). Cells were imaged on a DeltaVision Spectris wide field microscope fitted with a 37°C environment chamber (Solent Scientifi c, Segensworth, United Kingdom).

### Genomic sequencing

Genomic DNA from U2OS SEC-C and 2G TGFβ reporter single cell clones were isolated using DNeasy Blood & Tissue Kit (Qiagen). Genomic DNA was amplified by PCR using KOD Hot Start DNA Polymerase (Novagen). Primers used are as follows (5′-3′): PAI-1 genomic forward: CGCTGGGAAAGCATTAAGAG and PAI-1 genomic reverse: GAACTCCTAGCCTTGGGTGA. The PCR products were separated by size in 1% agarose gels. Genomic PCR of single cell clones positive for the 2G TGFβ reporter insertion by homologous recombination resulted in the amplification of a large PCR product of 5021 nucleotides (Allele-2) corresponding to the 2G TGFβ reporter insertion and a small fragment of 1850 nucleotides (Allele-1) corresponding to the non-recombined allele. The small PCR product of control and 2G reporter single cell clones were isolated with a gel extraction kit (Qiagen) and cloned into pSC-B-amp/kan Vector by using StrataClone Blunt PCR Cloning Kit (Agilent Technologies). For each genomic PCR cloning reaction, 20 positive colonies (white colonies) were amplified and DNA isolated by the DNA prep service at the Division of Signal Transduction Therapy (DSTT) at the University of Dundee. The cloning of genomic PCR fragments allows for the isolation and sequencing of the single DNA molecules corresponding to each allele. This procedure ensures the characterisation of the genotype of both alleles and avoids the possibility of cellular trisomy. We sequenced at least 20 clones of control and 2G TGFβ reporter isolated single cell lines. Primers used are as follows (5′-3′): AR906 forward: CACCTGCTTTTCTTCTAAGG and AR907 reverse: GGGAAGGGAGATGGACTGG.

### Real time quantitative reverse transcription PCR (qRT-PCR)

qRT-PCR experiments were performed as previously described^35^,^36^. Cells were seeded onto 6-well plates, serum starved for 16 h prior to TGFβ1 (50 pM) treatment. cDNA was made from 1 µg of the isolated RNA using the I-Script cDNA kit (BioRad) according to the manufacturer’s protocol. qRT-PCR reactions were performed in quadruplicate according to the manufacturer’s protocol in a CFX 384 Real time System qRT-PCR machine (BioRad). Each reaction included cDNA (2.5% of reverse transcriptase reaction) with forward and reverse primers (0.5 μM each) and 50% SYBR Green (BioRad). All primers were designed using PerlPrimer® and purchased from Invitrogen. Primers used are as follows (5′-3′): PAI-1 forward: AGCTCCTTGTACAGATGCCG, reverse: ACAACAGGAGGAGAAACCCA, GAPDH forward: TGCACCACCAACTGCTTAGC, reverse: GGCATGGACTGTGGTCATGAG, RPL13A forward: CCTGGAGGAGAAGAGGAAAGAGA, reverse: TTGAGGACCTCTGTGTATTTGTCAA. The primer efficiency was determined and taken into account when evaluating the qRT-PCR data. The data was normalised to the geometrical mean of two housekeeping genes (GAPDH and RPLI3A) and the Pfaffl method^37^ was used to analyse the qRT-PCR data.

### SDS-PAGE and immunoblotting

Reduced protein extracts (20  μg protein) were separated on 10% SDS-PAGE gels by electrophoresis and transferred to polyvinylidene fluoride membranes (Millipore). Membranes were blocked in 5% (w/v) non-fat milk in TBS-T (50  mM Tris-HCl pH 7.5, 150  mM NaCl, 0.2% Tween-20), incubated overnight at 4 °C in 5% milk-TBS-T or 3% BSA-TBS-T with the appropriate primary antibodies. After 3 x 10 min washes in TBS-T, membranes were incubated in horseradish peroxidase (HRP)-conjugated secondary antibodies diluted in 5% milk-TBS-T for 45 min at room temperature and washed further 3 x 10 min in TBS-T. Detection was performed by ECL luminescence (Thermo Scientific). The antibodies against phospho-Ser465/467 SMAD2 (#3101), total SMAD2/3 (#8658), SMAD4 (#9515), phospho-GSK3α/β (S21/9) (#9331) and GAPDH (#2118) were purchased from Cell Signaling. Anti-PAI-1 (ab66705) was from Abcam. Anti-TGFβR2 (sc-17792) was from Santa Cruz. Anti-Flag-M2 HRP (A8592) was from Sigma-Aldrich. Anti-tubulin (DM1A) was from Calbiochem. The anti-GFP and total anti-GSK3α/β antibodies were raised in sheep using recombinant proteins as antigen and affinity purified. Primary antibodies were used at 1:1000 dilution, except GAPDH, which was used at 1:5000 dilution. HRP-coupled secondary antibodies (1:5000) were obtained from Pierce.

### Southern blotting

Genomic DNA of U2OS SEC-C cell and TGFβ 2G Reporter clone-17 were isolated using DNeasy kit (Qiagen). 8 µg genomic DNA was digested with EcoR1 and separeted in a 1% agarose gel using a DNA molecular weight Digoxigenin-labeled (Roche). DNA was transfered to Hybond-N+ membrane (Amersham) in 10xSSC. Hybridization (50.8°C) and detection was perfomed according to manufacturer’s instructions in DIG Easy Hyb Kit (Roche) and DIG DNA detection Kit (Roche), respectivaly.

### Statistical analysis

All experiments have a minimum of three biological replicates. qRT-PCR experiments additionally have four technical repeats for each biological replicate. Data are presented as the mean with error bars indicating the standard deviation. Statistical significance of differences between experimental groups was assessed with Student’s t-test. Differences in means were considered significant if p<0.05. All Western blots shown are representatives.

## Author Contributions

ARF: Conception and design, acquisition of data, analysis and interpretation of data, drafting and revising the article. LH: Conception and design, acquisition of data, analysis and interpretation of data and drafting and revising the article. TM: Conception, design, cloning and contribution to unpublished essential data and reagents. CL: contributed the U2OS SEC-C system, and to unpublished essential data and reagents. RTH: Conception and design. GS: Conception and design, analysis and interpretation of data, drafting and revising the article.

## Supplementary Material

Supplementary Information Supplementary Information
