# Supplementary material for: Rapid generation of endogenously driven transcriptional reporters in cells through CRISPR/Cas9
Source: Sci Rep. 2015 Apr 29;5:9811. doi: 10.1038/srep09811 (PMC4413877; doi:10.1038/srep09811)
Supplement: Supplementary Information [file srep09811-s1.docx]

**Supplemental information index**

**Rapid generation of endogenously driven** **transcriptional reporters in cells through CRISPR/Cas9**

Alejandro Rojas-Fernandez^*1&3^, Lina Herhaus^*2^, Thomas Macartney^2^, Christophe Lachaud^2^, Ronald T. Hay^1^ and Gopal P. Sapkota^2&3^

^1^Centre for Gene Regulation and Expression, College of Life Sciences, University of Dundee, Dow Street, Dundee, DD1 5EH, Scotland, United Kingdom.

^2^MRC Protein Phosphorylation and Ubiquitylation Unit, College of Life Sciences, University of Dundee, Dow Street, Dundee, DD1 5EH, Scotland, United Kingdom.

^3^Address correspondence to Gopal P. Sapkota e-mail: g.sapkota@dundee.ac.uk & Alejandro Rojas-Fernandez e-mail: a.a.rojasfernandez@dundee.ac.uk

* These two authors contributed equally to this work.

Supplementary Figures legends

-Supplementary Figure 1

-Supplementary Figure 2

-Supplementary Figure 3

-Supplementary Figure 4

-Supplementary information file 1

**Supplementary Figure Legends**

**Supplementary Figure 1**

**a.** Schematic representation of the predicted genomic PCR products of the PAI-1 gene and 2G TGFβ reporter inserted allele. **b.** PCR products of genomic DNA of a fragment of the PAI-1 gene locus corresponding to the 2G TGFβ donor integration site. **c.** Frame shift on 2G TGFβ reporter cell line clone-17. The PAI-1 allele-1 has an insertion of one nucleotide indicated nucleotide leading to a frame-shift missense mutation. **d.** qRT-PCR of U2OS SEC-C control and 2G TGFβ reporter cell line clone normalized by control cell, indicate a decrease in the overall levels of PAI-1 mRNA. e) Schematic representation of wt and 2G donor insertion into the PAI-1 gene locus and the location of the Southern blot probe hybridazing to the firefly Luciferae cDNA. EcoR1 restriction sites flanking the region of inserion are highligted. f) Southern blotting analysis was perfomed using 8 µg of genomic DNA digested with EcoR1. A single recognition sequence was found in the clone-17 using a digoxigenin-labeled probe hybridizyng to the cDNA of firefly Luciferase, at the predicted size 4.9Kb. (*) non-specific binding.

**Supplementary Figure-2**

**a.** Knockdown efficiency was confirmed by immunoblotting using specific antibodies to SMAD4 and TGFBR2. **b.** Overexpression of FLAG-SMAD3 and GFP induction were confirmed by immunoblotting using a HRP conjugated anti FLAG antibody and a specific GFP antibody respectively. **c.** Activation of AP signalling upon PMA treatment was determined by immunoblotting with a GSP phospho-specific antibody.

**Supplementary Figure-3**

Outline of the time-scale required for the generation of a second-generation reporter (2G reporter).

**Supplementary Figure-4**

**a.** Schematic representation of the strategy used for the endogenous C-terminal YFP tagging of the Histone H3 Chaperone Chaf1a in U2OS SEC-C. **b.** Efficiency of YFP positive cell 7 days after sgRNA and Donor transfection. YFP positive cells were measured by FACS analysis.

Rojas-Fernandez_et_al._Supplementary_Figure-1

Rojas-Fernandez_et_al._Supplementary_Figure-2

Rojas-Fernandez_et_al._Supplementary_Figure-3

Rojas-Fernandez_et_al._Supplementary_Figure-4

Supplementary information file 1

**2G donor vector sequence**


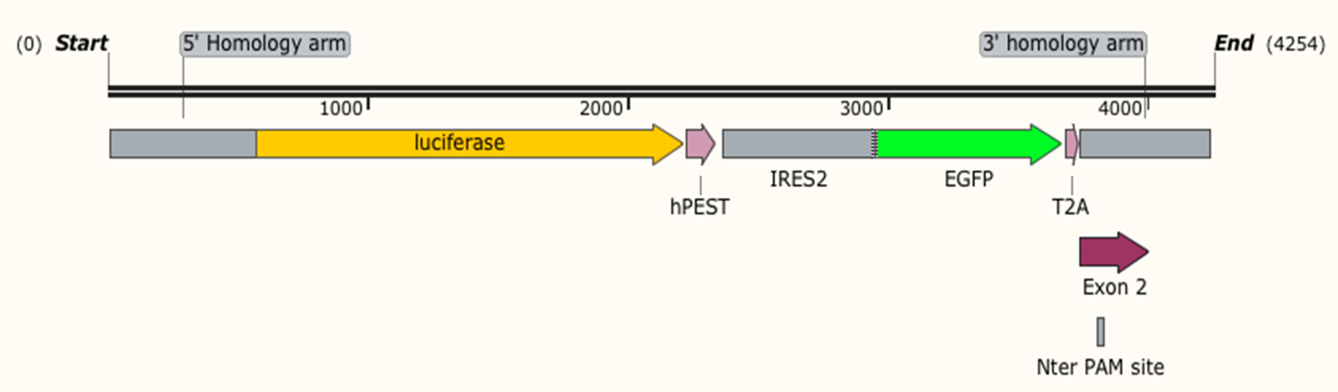


5’Homology arm

gcggccgcCCCTGTCTCTAAAAATGAATGAAAGGAAGGAAGAAAGAGAGAGAAAGAGAGAGAGGAAAGAAGGAAGGAAGTAAAGAAGAAAGAAAGAAAGAAAGAGGAAAGAGGAAGAAAGAAAGAAAAGAAAGAAAAGAAAAGAAAGCAAATTTAAAGCTTATGCAAATCAAAGATGTTGTGATAATTGATAATTGAGTCTGGGCTAAATTCCCCCTGGGCTGCAAAGGCAGAGAGTGGTAATGACTTCTCACCTGCTTTTCTTCTAAGGCTTTTTTACGGGACACAGAGGGAAGGGAGATGGACTGGATTCCAAGATTCCCACAGGGCAAGATGGGCGAAGACTCCCTGCCACTGCCCGGGGATAAGTCAGTCTGAGTGAGACGGAGTGGGATGGGCTTAGAACCTGAACATGTCATGGTCTCTTCCTGCACCTTGCCCTAGTGTTCACTTACCACCTGCTTGCAGGAAACAAGAAGAGCAGGGCCCACAGCTGGCCAGCTCCCCTCCCCTCCCGCCTGTCTTCCAGAACGATTCCTTCACCAGCCCTCTTTCCATTGCTCTAGG

Luciferase

ATGGAAGATGCCAAAAACATTAAGAAGGGCCCAGCGCCATTCTACCCACTCGAAGACGGGACCGCCGGCGAGCAGCTGCACAAAGCCATGAAGCGCTACGCCCTGGTGCCCGGCACCATCGCCTTTACCGACGCACATATCGAGGTGGACATTACCTACGCCGAGTACTTCGAGATGAGCGTTCGGCTGGCAGAAGCTATGAAGCGCTATGGGCTGAATACAAACCATCGGATCGTGGTGTGCAGCGAGAATAGCTTGCAGTTCTTCATGCCCGTGTTGGGTGCCCTGTTCATCGGTGTGGCTGTGGCCCCAGCTAACGACATCTACAACGAGCGCGAGCTGCTGAACAGCATGGGCATCAGCCAGCCCACCGTCGTATTCGTGAGCAAGAAAGGGCTGCAAAAGATCCTCAACGTGCAAAAGAAGCTACCGATCATACAAAAGATCATCATCATGGATAGCAAGACCGACTACCAGGGCTTCCAAAGCATGTACACCTTCGTGACTTCCCATTTGCCACCCGGCTTCAACGAGTACGACTTCGTGCCCGAGAGCTTCGACCGGGACAAAACCATCGCCCTGATCATGAACAGTAGTGGCAGTACCGGATTGCCCAAGGGCGTAGCCCTACCGCACCGCACCGCTTGTGTCCGATTCAGTCATGCCCGCGACCCCATCTTCGGCAACCAGATCATCCCCGACACCGCTATCCTCAGCGTGGTGCCATTTCACCACGGCTTCGGCATGTTCACCACGCTGGGCTACTTGATCTGCGGCTTTCGGGTCGTGCTCATGTACCGCTTCGAGGAGGAGCTATTCTTGCGCAGCTTGCAAGACTATAAGATTCAATCTGCCCTGCTGGTGCCCACACTATTTAGCTTCTTCGCTAAGAGCACTCTCATCGACAAGTACGACCTAAGCAACTTGCACGAGATCGCCAGCGGCGGGGCGCCGCTCAGCAAGGAGGTAGGTGAGGCCGTGGCCAAACGCTTCCACCTACCAGGCATCCGCCAGGGCTACGGCCTGACAGAAACAACCAGCGCCATTCTGATCACCCCCGAAGGGGACGACAAGCCTGGCGCAGTAGGCAAGGTGGTGCCCTTCTTCGAGGCTAAGGTGGTGGACTTGGACACCGGTAAGACACTGGGTGTGAACCAGCGCGGCGAGCTGTGCGTCCGTGGCCCCATGATCATGAGCGGCTACGTTAACAACCCCGAGGCTACAAACGCTCTCATCGACAAGGACGGCTGGCTGCACAGCGGCGACATCGCCTACTGGGACGAGGACGAGCACTTCTTCATCGTGGACCGGCTGAAGAGCCTGATCAAATACAAGGGCTACCAGGTAGCCCCAGCCGAACTGGAGAGCATCCTGCTGCAACACCCCAACATCTTCGACGCCGGGGTCGCCGGCCTGCCCGACGACGATGCCGGCGAGCTGCCCGCCGCAGTCGTCGTGCTGGAACACGGTAAAACCATGACCGAGAAGGAGATCGTGGACTATGTGGCCAGCCAGGTTACAACCGCCAAGAAGCTGCGCGGTGGTGTTGTGTTCGTGGACGAGGTGCCTAAAGGACTGACCGGCAAGTTGGACGCCCGCAAGATCCGCGAGATTCTCATTAAGGCCAAGAAGGGCGGCAAGATCGCCGTGACTAGT

hPEST

TCTCACGGCTTCCCTCCCGAGGTGGAGGAGCAGGCCGCCGGCACCCTGCCCATGAGCTGCGCCCAGGAGAGCGGCATGGATAGACACCCTGCTGCTTGCGCCAGCGCCAGGATCAACGTCT

IRES2

AAGGTACCGCGGGCCCGGGATCCGCCCCTCTCCCTCCCCCCCCCCTAACGTTACTGGCCGAAGCCGCTTGGAATAAGGCCGGTGTGCGTTTGTCTATATGTTATTTTCCACCATATTGCCGTCTTTTGGCAATGTGAGGGCCCGGAAACCTGGCCCTGTCTTCTTGACGAGCATTCCTAGGGGTCTTTCCCCTCTCGCCAAAGGAATGCAAGGTCTGTTGAATGTCGTGAAGGAAGCAGTTCCTCTGGAAGCTTCTTGAAGACAAACAACGTCTGTAGCGACCCTTTGCAGGCAGCGGAACCCCCCACCTGGCGACAGGTGCCTCTGCGGCCAAAAGCCACGTGTATAAGATACACCTGCAAAGGCGGCACAACCCCAGTGCCACGTTGTGAGTTGGATAGTTGTGGAAAGAGTCAAATGGCTCTCCTCAAGCGTATTCAACAAGGGGCTGAAGGATGCCCAGAAGGTACCCCATTGTATGGGATCTGATCTGGGGCCTCGGTGCACATGCTTTACATGTGTTTAGTCGAGGTTAAAAAAACGTCTAGGCCCCCCGAACCACGGGGACGTGGTTTTCCTTTGAAAAACACGATGATAATATGGCCACAACC

eGFP

ATGGTGAGCAAGGGCGAGGAGCTGTTCACCGGGGTGGTGCCCATCCTGGTCGAGCTGGACGGCGACGTAAACGGCCACAAGTTCAGCGTGTCCGGCGAGGGCGAGGGCGATGCCACCTACGGCAAGCTGACCCTGAAGTTCATCTGCACCACCGGCAAGCTGCCCGTGCCCTGGCCCACCCTCGTGACCACCCTGACCTACGGCGTGCAGTGCTTCAGCCGCTACCCCGACCACATGAAGCAGCACGACTTCTTCAAGTCCGCCATGCCCGAAGGCTACGTCCAGGAGCGCACCATCTTCTTCAAGGACGACGGCAACTACAAGACCCGCGCCGAGGTGAAGTTCGAGGGCGACACCCTGGTGAACCGCATCGAGCTGAAGGGCATCGACTTCAAGGAGGACGGCAACATCCTGGGGCACAAGCTGGAGTACAACTACAACAGCCACAACGTCTATATCATGGCCGACAAGCAGAAGAACGGCATCAAGGTGAACTTCAAGATCCGCCACAACATCGAGGACGGCAGCGTGCAGCTCGCCGACCACTACCAGCAGAACACCCCCATCGGCGACGGCCCCGTGCTGCTGCCCGACAACCACTACCTGAGCACCCAGTCCGCCCTGAGCAAAGACCCCAACGAGAAGCGCGATCACATGGTCCTGCTGGAGTTCGTGACCGCCGCCGGGATCACTCTCGGCATGGACGAGCTGTACAAGTCCGGACTCGGATCC

T2A

GAGGGCAGAGGAAGTCTTCTAACATGCGGTGACGTGGAGGAGAATCCCGGCCCA

3’ homology arm including Exon 2 (red) – silent Pam KO mutations lower case

CAGATGTCTCCAGCCCTCACCTGCCTAGTCCTGGGCCTGGCCCTTGTCTTTGGTGAAGGGTCTGCTGTaCAtCAcCCtCCgagtTAtGTaGCCCACCTGGCCTCAGACTTCGGGGTGAGGGTGTTTCAGCAGGTGGCGCAGGCCTCCAAGGACCGCAACGTGGTTTTCTCACCCTATGGGGTGGCCTCGGTGTTGGCCATGCTCCAGCTGACAACAGGAGGAGAAACCCAGCAGCAGATTCAAGCAGCTATGGGATTCAAGATTGATGGTGAGCCACGGGACACCAGGGGAGGTGGGTGGCATGCAGAACAGACCTACCAGAAGCCAAGGAAAGGCTGGCTCTGGCTTAGCCGAGCCAAGCCCCATACAGCTGTGCTGCAGGGGCCACCCCATCTTCTTCCCACTACACTCCAAGTCACTGGACCCTTGAATCTCCAAGGGTGTCTGACCAGTAGATTTACCGCTTATTCACCACCGTGTGATCTTAACCTCGTTAAGTTgcggccgc

**Southern Blot Information**

Probe

**GAAGGGCCCAGCGCCATTC**TACCCACTCGAAGACGGGACCGCCGGCGAGCAGCTGCACAAAGCCATGAAGCGCTACGCCCTGGTGCCCGGCACCATCGCCTTTACCGACGCACATATCGAGGTGGACATTACCTACGCCGAGTACTTCGAGATGAGCGTTCGGCTGGCAGAAGCTATGAAGCGCTATGGGCTGAATACAAACCATCGGATCGTGGTGTGCAGCGAGAATAGCTTGCAGTTCTTCATGCCCGTGTTGGGTGCCCTGTTCATCGGTGTGGCTGTGGCCCCAGCTAACGACATCTACAACGAGCGCGAGCTGCTGAACAGCATGGGCATCAGCCAGCCCACCGTCGTATTCGTGAGCAAGAAAGGGCTGCAAAAGATCCTCAACGTGCAAAAGAAGCTACCGATCATACAAAAGATCATCATCATGGATAGCAAGACCGACTACCAGGGCTTCCAAAGCATGTACACCTTCGTGACTTCCCATTTGCCACCCGGCTTCAACGAGTACGACTTCGTGCCCGAGAGCTTCGACCGGGACAAAACCATCGCCCTGATCATGAACAGTAGTGGCAGTACCGGATTGCCCAAGGGCGTAGCCCTACCGCACCGCACCGCTTGTGTCCGATTCAGTCATGCCCGCGACCCCATCTTCGGCAACCAGATCATCCCCGACACCGCTATCCTCAGCGTGGTGCCATTTCACCACGGCTTCGGCATGTTCACCACGCTGGGCTACTTGATCTGCGGCTTTCGGGTCGTGCTCATGTACCGCTTCGAGGAGGAGCTATTCTTGCGCAGCTTGCAAGACTATAAGATTCAATCTGCCCTGCTGGTGCCCACACTATTTAGCTTCTTCGCTAAGAGCACTCTCATCGACAAGTACGACCTAAGCAACTTGCACGAGATCGCCAGCGGCGGGGCGCCGCTCAGCAAGGAGGTAGGTGAGGCCGTGGCCAAACGCTTCCACCTACCAGGCATCCGCCAGGGCTACGGCCTGACAGAAACAACCAGCGCCATTCTGATCACCCCCGAAGGGGACGACAAGCCTGGCGCAGTAGGCAAGGTGGTGCCCTTCTTCGAGGCTAAGGTGGTGGACTTGGACACCGGTAAGACACTGGGTGTGAACCAGCGCGGCGAGCTGTGCGTCCGTGGCCCCATGATCATGAGCGGCTACGTTAACAACCCCGAGGCTACAAACGCTCTCATCGACAAGGACGGCTGGCTGCACAGCGGCGACATCGCCTACTGGGACGAGGACGAGCACTTCTTCATCGTGGACCGGCTGAAGAGCCTGATCAAATACAAGGGCTACCAGGTAGCCCCAGCCGAACTGGAGAGCATCCTGCTGCAACACCCCAACATCTTCGACGCCGGGGTCGCCGGCCTGCCCGACGACGATGCCGGCGAGCTGCCCGCCGCAGTCGTCGTGCTGGAACACGGTAAAACCATGACCGAGAAGGAGATCGTGGACTATGTGGCCAGCCAGGTTACAACCGCCAAGAAGCTGCGCGGTGGTGTTGTGTTCGTGGACGAGGTGCCTAAAGGACTGA**CCGGCAAGTTGGACGCCCGC**

Primers

**Forward: 5’ GAAGGGCCCAGCGCCATTC 3’**

**Reverse: 5’ GCGGGCGTCCAACTTGCCGG 3’**
